# Stress, interpersonal and inter-role conflicts, and psychological health conditions among nurses: vicious and virtuous circles within and beyond the wards

**DOI:** 10.1186/s40359-024-01676-y

**Published:** 2024-04-10

**Authors:** Federica Vallone, Maria Clelia Zurlo

**Affiliations:** 1 Department of Humanities, University of Naples Federico II, Naples, Italy; 2 Dynamic Psychology Laboratory, University of Naples Federico II, Via Rodinò 22, Naples, Italy

**Keywords:** Interpersonal conflicts, Inter-role conflict, Hostility, Interpersonal-sensitivity, Nursing, Psychological health, Work-related stress

## Abstract

**Background:**

The increasing costs of nurses’ occupational-stress, conflicts, and violence within healthcare services have raised international interest. Yet, research/interventions should consider that perceived stress and conflicts– but also potential resources– within the wards can crossover the healthcare settings, impacting nurses’ private lives and *viceversa*, potentially creating vicious circles exacerbating stress, conflicts/violence or, conversely, virtuous circles of psychological/relational wellbeing. Based on the *Demands-Resources-and-Individual-Effects (DRIVE) Nurses Model*, and responding to the need to go in-depth into this complex dynamic, this study aims to explore potential vicious circles featured by the negative effects of the interplay (main/mediating effects) between perceived stressors in nursing linked to interpersonal conflicts (Conflicts-with-Physicians, Peers, Supervisors, Patients/their families), work-family inter-role conflicts (Work-Family/Family-Work-Conflicts), and work-related stress (Effort-Reward-Imbalance) on nurses’ psychological/relational health (Anxiety, Depression, Somatization, Interpersonal-Sensitivity, Hostility). The potential moderating role of work-resources (Job-Control, Social-Support, Job-Satisfaction) in breaking vicious circles/promoting virtuous circles was also explored.

**Method:**

The STROBE Checklist was used to report this cross-sectional multi-centre study. Overall, 265 nurses completed self-report questionnaires. Main/mediating/moderating hypotheses were tested by using Correlational-Analyses and Hayes-PROCESS-tool.

**Results:**

Data confirmed the hypothesized detrimental vicious circles (main/mediating effects), impairing nurses’ psychological health conditions at individual level (Anxiety, Depression, Somatization), but also at relational level (Hostility and Interpersonal-Sensitivity). The moderating role of all work resources was fully supported.

**Conclusion:**

Findings could be used to implement interventions/practices to effectively prevent the maintenance/exacerbation of vicious circles and promote psychological/relational wellbeing in healthcare settings and beyond.

**Supplementary Information:**

The online version contains supplementary material available at 10.1186/s40359-024-01676-y.

Promoting occupational health and safety in the healthcare sector is a serious concern globally, and it is still at the heart of the international debate [1, 2]. Specifically, in recent decades, growing concern and attention have been given to nursing professionals [3, 4], who are required to achieve gold standard with fewer resources (e.g., staff shortage), to perform more of the daily care activities in direct contact with patients/their relatives, as well as to coordinate– to a greater extent– with colleagues (other nurses and physicians). This results in higher risk of perceiving imbalance between expended efforts and received rewards/recognitions for their work [5, 6] and feeling more emotionally exhausted than other healthcare professionals [7, 8].

Recently, responding to the need to provide a tailored tool to assess work-related stress and develop effective interventions promoting occupational health among nursing professionals, research has provided a statistically valid multidimensional transactional model [9]. Based on the *Demands-Resources and Individual Effects Model* (*DRIVE Model*) [10–12], this model, namely the *DRIVE-Nurses Model*, allows to simultaneously account for the impact of the complex interplay (main, mediating, moderating effects) of a wide range of individual, situational, and relational dimensions to be adopted in the public healthcare services for a broad assessment of risks and protective factors influencing nurses’ psychological and physical health conditions [9]. Specifically, the model includes the following dimensions: *Work Characteristics*– integrating Effort-Reward Imbalance Model dimensions [13] and Job Demands-Control-Support Model dimensions [14]– along with *Individual Characteristics* (i.e., socio-demographics, coping strategies, personality factors), and *Work–Family Interface Dimensions* (i.e., Work–family inter-role conflict; job/life satisfaction).

Noticeably, the abovementioned model fully embodies the more renowned perspective for achieving a greater and more comprehensive understanding of workers’ life, namely the *Work-Family Spillover* perspective [15, 16]. The latter approach reconceptualises the complexity of the workers’ lives, extending to the understanding of individual’s life. It suggests that research/interventions need to take into account that the positive and the negative feelings/experiences in one domain (workplace or family/private) are able to crossover their boundaries, having a positive (enrichment) or negative (conflict) impact also on the other one domain.

In line with this, recent research applications of the *DRIVE-Nurses Model* specifically targeted the examination of perceived inter-role conflict among nurses [17]. In particular, evidence was provided for the detrimental impact of perceived work-family conflict among both male and female nurses, yet it was also supported the moderating role of work-resources, such as perceived job control, social support, and job satisfaction. These resources were indeed found able to significantly buffer/counteract the negative effects of perceived inter-role conflict among nurses.

Nowadays, besides the meaningful of focussing on perceived conflict between work and private life, and in line with the *spillover perspective*, research highlighted another key issue that needs to be carefully targeted globally, namely perceived conflict and violence within the healthcare settings [18–20]. Indeed, about 63% of healthcare workers worldwide reported they have experienced any form of violence and conflict within the workplace [2, 21], including verbal abuses, such as threatening/bullying/offending/excluding behaviours (e.g., shouting, insults, overload in work shifts), and/or physical abuses (e.g., assaults/aggressions, attempted assaults/aggressions).

This already high rate should however be evaluated with caution, since episodes of violence– mainly linked to exacerbation of conflicts and non-physical abuses– are still often not reported, underreported and/or partially documented by nurses, mainly due to the stigma of victimization (i.e., shame, fear of judgement, blame/non-supportive environment), fear of consequences for their selves/fear of lack of consequences for the perpetrators, as well as due to the lack of knowledge about the reporting process/systems [22, 23]. Furthermore, there is still lack of knowledge and clarity on what constitute a violence, thus verbal abuses and exacerbation of interpersonal conflicts are often not reported since they are underestimated and believed as not enough serious to seek for support [24–26].

Notwithstanding, in recent decades, research targeted this key topic, underlining the need to identify both the potentially different actors/critical relations involved onto conflictual/violent dynamics (i.e., stressors linked to interpersonal conflict) and the consequences of such issue [27–30]. Indeed, several studies have underlined not only nurses’ risk of reporting decreasing performance, lower quality of care, and higher turnover intention [31–34], but also the severe risk for nurses’ psychological health conditions, in terms of anxiety, depression [35–38], and somatization [39, 40]. Some studies have also underlined the impact of perceived workplace violence in terms of increasing frustration, disruptions in the relationships with co-workers [41], inappropriate professional communication [4], and growing hostility [32, 34, 42], with even some evidence suggesting the risk of a spiral effect of violence and interpersonal conflict [43].

Nonetheless, despite the well-demonstrated worrisome high prevalence of perceived occupational stress, violence/conflicts towards and between nurses, and the growing implementation of programmes targeting this issue, there is still lack of research focusing on this phenomenon by adopting a more comprehensive and dynamic approach. This, however, could allow gaining further insight into the complex relationship between perceived stress, conflicts and psychological/relational wellbeing experienced within and outside the wards. Indeed, the interplay between work and private lives is rather complex and tangled [44]. Accordingly, perceived sources of stress and conflicts– along with the potential resources– within the wards are able to crossover the healthcare settings, impacting nurses’ personal lives and *viceversa*, thus potentially creating vicious circles exacerbating stress, conflicts, imbalances, psychological suffering, anger and hostility or, conversely, sustaining virtuous circles of psychological and relational wellbeing.

Therefore, there is a need to provide updated evidence which based on exploring in-depth more complex dynamics featuring the reciprocity of the experiences within the wards and outside the wards. Additionally, there is a need to investigate the impact of workplace violence and conflicts by assessing the risk of reporting a wider range of potential outcomes, such as interpersonal-sensitivity and hostility, which are of particular interest when examining interpersonal relationships. Unveiling vicious and virtuous circles could indeed effectively inform the development of tailored research and evidence-based interventions.

## Objective

The present study proposed a research application of the *DRIVE-Nurses Model* [9] to achieve a more comprehensive understanding of the issue of stress, violence and conflictual dynamics in nursing. In the present paper, we will analyse the issue of workplace violence and conflict by assessing perceived stressors linked to interpersonal conflict in nursing [45]– namely perceived stress linked to problems/conflicts with physicians, with peers, with supervisors as well as with patients and their families– rather than by assessing the frequency of episodes of violence/conflicts occurred. This choice was made given the phenomena of underreported/underestimated events of violence and conflicts– mainly non-physical ones [22–26]. It was indeed hoped that focusing on perceived problems and conflicts in the interpersonal life of nurses– rather than assessing events of violence within these relationships– would elicit a more open reflection on this hidden phenomenon, giving further research attention to the more subtle forms of experiences of violence in nursing.

**Fig. 1:**
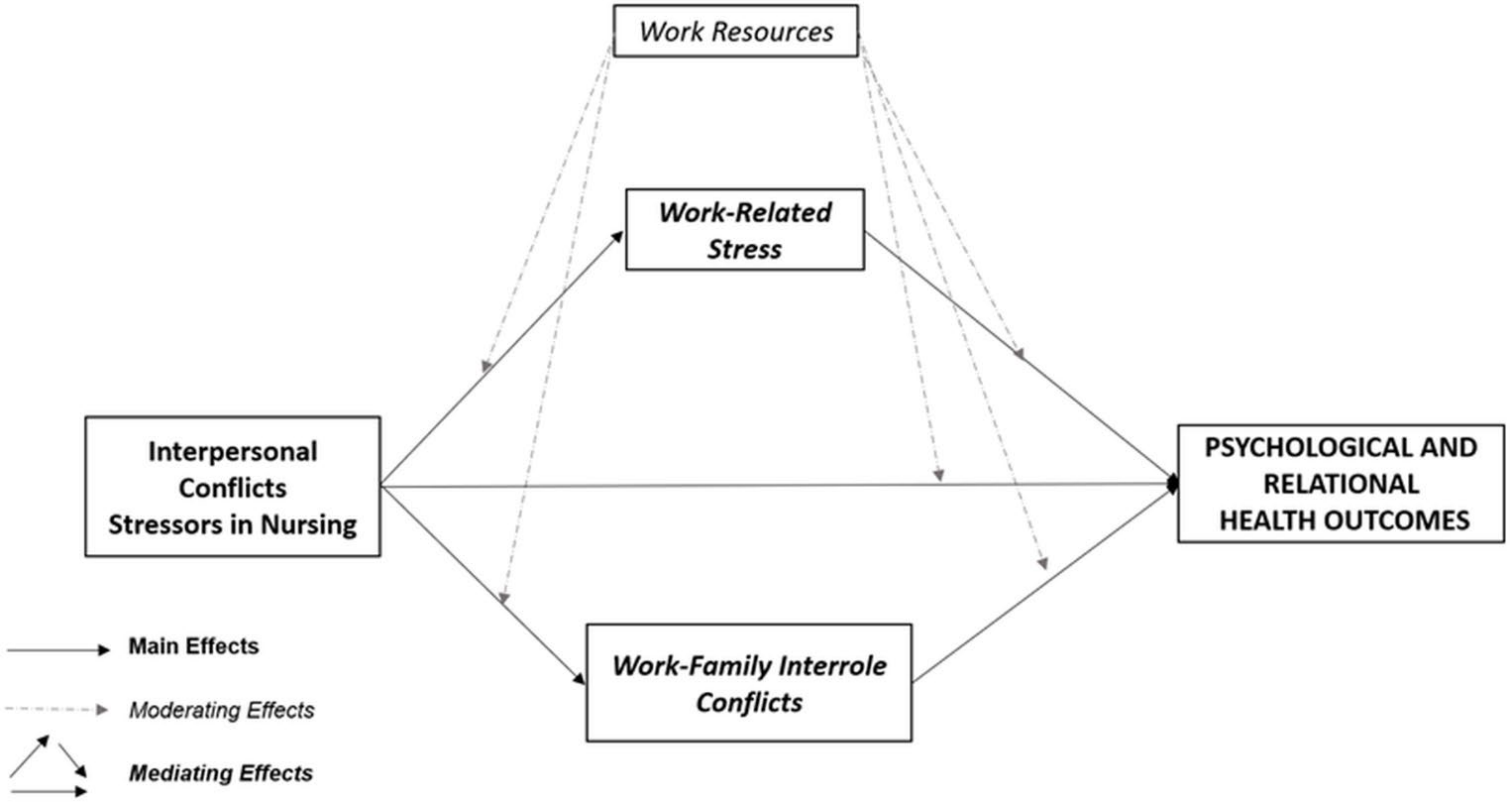
Conceptual Framework: Main, Mediating and Moderating Hypotheses

Specifically, the study has a twofold objective in mind: (1) To explore potential vicious circles featured by the negative effects of the interplay (main/mediating effects) between perceived stressors in nursing linked to interpersonal conflicts (Conflicts-with-Physicians, Peers, Supervisors, Patients/their families), work-family inter-role conflicts (Work-Family/Family-Work Conflicts), and work-related stress dimensions (Effort-Reward-Imbalance) on nurses’ psychological and relational health conditions (Anxiety, Depression, Somatization, Interpersonal-Sensitivity, Hostility); (2) To test the potential moderating role of work resources (Job-Control, Social-Support, Job-Satisfaction) in breaking vicious circles/promoting virtuous circles. Accordingly, detailed hypotheses have been developed and tested (Fig. 1).

Firstly, considering the evidence suggesting the detrimental impact of workplace violence and conflicts [4, 34–36, 39, 41, 46, 47], of work-family inter-role conflict [4, 17], as well as of work-related stress [9, 48–52] on nurses’ psychological and relational health conditions, the following hypothesis was proposed:

*Hypothesis one* (H1)– *Main Effects*. Perceived stressors in nursing linked to interpersonal conflicts (H1a), work-family inter-role conflicts (H1b), and work-related stress (H1c) dimensions will be positively related to nurses’ psychological and relational health conditions.

Secondly, considering the evidence suggesting the potential of perceived workplace conflict/violence in exacerbating perceived levels of work-family inter-role conflicts [4, 53], and work-related stress [33, 54–56], as well as the potential of vicious circles of stress and conflicts exacerbating psychological and relational suffering [43], the following hypothesis was tested.

*Hypothesis two* (H2)– *Mediating Effects*. Perceived stressors in nursing linked to interpersonal conflicts will be positively related to work-family inter-role conflicts (H2a) and to work-related stress (H2b) dimensions. In addition, work-family inter-role conflicts (H2c) and work-related stress (H2d) dimensions will play as mediators in the associations between stressors in nursing linked to interpersonal conflicts and nurses’ psychological and relational health conditions.

Finally, considering the well-demonstrated protective role of work-resources [9, 17] and, in particular, the moderating role of perceived control [57, 58], organizational support [59–61], and job satisfaction [62, 63], the following hypothesis was tested:

*Hypothesis three* (H3)– *Moderating Effects*. Perceived Work-Resources, namely Job Control (H3a), Social Support (H3b), and Job Satisfaction (H3c) will significantly moderate/buffer the relationships between perceived stressors in nursing linked to interpersonal conflicts and, respectively, work-family inter-role conflicts, work-related stress, and psychological and relational health conditions. In addition, Work-Resources will also significantly moderate/buffer the relationships between, respectively, work-family inter-role conflicts and work-related stress with psychological and relational health conditions.

## Materials and methods

### Sample

The present cross-sectional multi-centre study was carried out in a sample of 265 nurses, recruited from Italian Hospitals of the Public Health Service between January and August 2023. The STROBE Checklist was used to report this study. Chairpersons and– where available– Nursing Managers were contacted to ask for the permission for administering a questionnaire to the nursing staff (in-presence administration, with a trained psychologist always available to respond to any doubt/queries). Afterwards, nurses were directly contacted and given all the information about the research objective as well as about the confidentiality of the data collection procedure. To be included, participants need to be nurses working in Public Health Service (i.e., high-specialized hospital, academic hospital, general hospital). Those nurses working in private settings were not covered in the present study. The study was performed in accordance with the 1964 Helsinki Declaration and its later amendments or comparable ethical standards, and it was approved by the Ethics Committee of Psychological Research of University of Naples Federico II. A total of 265 out of 300 nurses agreed to participate in the study, provided the informed consent, and completed the questionnaire in all its section (Response Rate = 88.3%). There were no missing data. Overall, the sample is representative of the diverse nursing workforce for sex and age. Indeed, 39.6% (*n* = 105) were men, 60.7% (*n* = 160) were women, and the ages ranged from 21 to 65 years (*M* = 44.3, *SD* = 10.0).

### Measures

The questionnaire included a section for registering nurses’ background information, along with validated measures for the assessment of perceived stressors in nursing linked to interpersonal conflicts, work-family inter-role conflicts, work-related stress, work-resources, and psychological and relational health conditions.

### Stressors in nursing-interpersonal conflicts

Perceived stress linked to interpersonal conflicts was assessed by using the Expanded Nursing Stress Scale (ENSS) [45], which consists of 57 items on a 5-point Likert scale (ranging from 1 = “never stressful” to 4 = “extremely stressful”, with 0 = “does not apply”) and divided into nine subscales, namely Death and Dying, Inadequate Emotional Preparation, Discrimination, Uncertainty Concerning Treatments, Workload, Conflicts with Physicians, Conflicts with Peers, Conflicts with Supervisors, Patients and their Families. In line with our research objectives, four subscales out of nine of the ENSS were used, namely Conflicts with Physicians (5 items, e.g., “Criticism by a physician”); Conflicts with Peers (6 items; e.g., “Difficulty with another nurse in immediate work setting”); Conflicts with Supervisors (7 items, e.g., “Criticisms by a supervisor”); and Patients and their Families (8 items, e.g., “Dealing with abusive patients”). In the present study, Cronbach’s α and McDonald’s ω values were satisfactory (Supplementary Table 1).

### Work-family inter-role conflicts

Perceived work–family inter-role conflicts were assessed by using the Work–Family Conflict and Family–Work Conflict Scales (WFC and FWC) [64, 65]. Each scale (WFC; e.g., “My job produces strain that makes it difficult to fulfil family duties”; FWC, e.g., “The demands of my family or spouse/partner interfere with work-related activities”) consists of 5 items on a 7-point Likert scale (ranging from 1 = “Strongly disagree” to 7 = “Strongly agree”). In this study, Cronbach’s α and McDonald’s ω values were satisfactory (Supplementary Table 1).

### Work-related stress

Perceived work-related stress was assessed by using the Effort-Reward Imbalance Test (ERI Test) [13, 66], which consists of 17 items on a 5-point Likert scale (ranging from 1 = “Disagree” to 5 = “Agree, and I am very distressed”) divided into three subscales, namely Effort (6 items, e.g., “Over the past few years, my job has become more and more demanding”), Material Reward (7 items, e.g., “Considering all my efforts and achievement, my salary/income is adequate”) and Esteem Reward (4 items, e.g., “I received the respect I deserve from my superiors”). In line with the ERI Model [13], in the present study, we have adopted the Effort-Reward Imbalance (ERI) ratio, which represents the ratio of perceived Effort (numerator) and perceived Rewards (denominator). ERI ratio can be calculated by using the following formula i.e., Effort score/Total Rewards score multiplied by a correction factor derived from the difference in the number of items for Effort and Rewards. ERI ratio increases with increasing values of the ratio, with cut-off score of 1 (ERI ratio > 1) indicating high levels of perceived imbalance [13, 67]. In the present study, Cronbach’s α and McDonald’s ω values for Effort and Total Reward scales were satisfactory (Supplementary Table 1).

### Work-resources

Perceived work resources were assessed by using the Job Content Questionnaire (JCQ) [14] and the Job Satisfaction Subscale from the Copenhagen Psychosocial Questionnaire (COPSOQ) [68].

The Job Content Questionnaire (JCQ) [14] consists of 27 items on a four-point Likert scale (ranging from 0 = “Often” to 3 = “Never”) divided into three subscales, namely Job Demands, Job Control, and Social Support. In the present study, we used the two subscales of job control (14 items, e.g., “Do you have a choice in deciding how you do your work?”), and social support (4 items, e.g., “How often do you get help and support from your immediate superior?”). The Job Satisfaction subscale from the Copenhagen Psychosocial Questionnaire (COPSOQ) [68] consists of four items on a 4-point Likert scale (ranging from 0 = “Highly unsatisfied” to 3 = “Very satisfied”), covering perceived satisfaction in the form of working conditions, perspectives and usage of abilities (4 items, e.g., Regarding your work in general, how pleased are you with your work prospects?). In the present study, Cronbach’s α and McDonald’s ω values for all Work-Resources were satisfactory (Supplementary Table 1).

### Psychological and relational health conditions

Perceived levels of psychological and relational health conditions were assessed by using the Symptom Checklist-90-Revised (SCL-90-R) [69, 70], which consists of 90 items on a five-point Likert scale (ranging from 0 = “Not at all” to 4 = “Extremely”), and divided into nine subscales, namely Anxiety, Phobic-Anxiety, Obsessive–Compulsive, Somatization, Depression, Interpersonal-Sensitivity, Hostility, Psychoticism, and Paranoid Ideation. In line with our research objectives, four subscales out of nine of the SCL-90-R were used, namely Anxiety (10 items, e.g., “Tense or keyed up”), Depression (13 items, e.g., “Hopeless about future”), Somatization (12 items, e.g., “Feeling weak”), Interpersonal-Sensitivity (9 items, e.g., “Feeling that people are unfriendly or dislike you”), and Hostility (6 items, e.g., “Having urges to break or smash things”). Additionally, in order to identify nurses reporting clinically relevant levels of symptoms, scores were also converted into percentages by using the cut-off points provided in the Italian validation study [70] according to age (adults) and to sex (i.e., Anxiety: cut-off men = 0.91, women = 1.31; Depression: cut-off men = 1.08, women = 1.62; Somatization: cut-off men = 1.09, women = 1.67; Interpersonal-Sensitivity: cut-off men = 1.01, women = 1.34; Hostility: cut-off men = 1.18, women = 1.34).

### Data analysis

All the statistical analyses were carried out by using the Statistical Package for the Social Sciences (SPSS; Version 21). Firstly, descriptive statistics of study variables were carried out, and frequencies and percentages of nurses reporting clinically relevant levels of symptoms of Anxiety, Depression, Somatization, Interpersonal-Sensitivity, and Hostility were calculated by using the cut-off scores provided by the Italian validation study of the SCL-90-R [70]. Moreover, preliminary to mediating and moderating hypotheses testing, Correlational Analyses (i.e., Pearson’s correlations among all study variables) were undertaken to explore correlations between, respectively, stressors in nursing linked to interpersonal conflicts, inter-role conflicts and work-related stress dimensions with psychological and relational health outcomes (H1), as well as to evaluate the feasibility of the testing of mediating (H2) and moderating hypotheses (H3). Furthermore, to judge the normality of data, the distribution of variables was explored by calculating Skewness (*range* − 2 to + 2) and Kurtosis values (*range* − 7 to + 7) (i.e., Skewness = 2 and kurtosis = 7 considered to be a violation of normality) [71–74].

Therefore, in order to test mediating (H2) and moderating (H3) hypotheses, Hayes’ PROCESS tool was used (Model 4 for mediation analyses and Model 1 for moderation analyses) [75]. For analysing and reporting direct and indirect (mediation) analyses, bias-corrected bootstrapped test with 5,000 replications to ensure the 95% Confidence Interval (Confident Interval with the lower and the upper bounds either both positive or both negative) were used to verify the significance of the effects [75], while the Z Sobel test was used to ensure the significance of indirect effects (*Z* > 1.96, *p* <.05). For analysing and reporting moderation analyses, the statistical significance of interaction effects was examined (*p* <.05), the delta R-sq values (ΔR^2^) were reported to display that the inclusion of the interaction terms resulted in a statistically significant increase in the variance explained in the outcomes. Finally, simple slopes were plotted to graphically display moderating effects. The Variance Inflation Factor (VIF) and tolerance values were also used for diagnosing multicollinearity, using VIF < 5 and tolerance > 0.40 as cut-off points [76, 77].

## Results

### Preliminary analyses

With respect to psychological and relational health conditions, data showed that 18.5% (*n* = 49) of nurses reported clinically relevant levels of Somatization, 15.5% (*n* = 41) clinical levels of Depression, and 14.0% (*n* = 37) reported clinical levels of Anxiety. Moreover, 18.1% (*n* = 48) of nurses reported clinical levels of Interpersonal-Sensitivity and 11.7% (*n* = 31) showed clinical levels of Hostility.

### Hypothesis one (H1)– main effects

Table 1 shows means, standard deviations and inter-correlations among study variables.

**Table 1:** Mean scores, Standard Deviations, and Intecorrelations among the study variables (*n* = 265)

	M	SD	1	2	3	4	5	6	7	8	9	10	11	12	13	14	15
**Interpersonal Conflicts Stressors in Nursing**																	
1. Conflicts with Physician	8.66	4.87	1														
2. Conflicts with Peers	7.83	5.11	0.68^**^	1													
3. Conflicts wirh Supevisors	13.14	6.74	0.58^**^	0.47^**^	1												
4. Patients and their Families	16.86	8.20	0.77^**^	0.51^**^	0.64^**^	1											
**Work-Related Stress**																	
5. Effort-Reward Imbalance	0.79	0.44	0.09	0.11	0.15^*^	0.06	1										
**Work-Family Inter-role Conflicts**																	
6. Work-Family Conflict	18.38	7.79	0.25^**^	0.17^**^	0.22^**^	0.24^**^	0.32^**^	1									
7. Family-Work Conflict	14.27	7.29	0.18^**^	0.07	0.08	0.11	0.03	0.57^**^	1								
**Work Resources**																	
8. Job Control	35.76	6.37	− 0.07	− 0.10	− 0.10	− 0.1	− 0.17^**^	− 0.19^**^	− 0.13^*^	1							
9. Social Support	11.03	3.24	− 0.04	− 0.04	− 0.01	− 0.02	− 0.23^**^	− 0.08	− 0.06	0.19^**^	1						
10. Job Satisfaction	10.38	2.98	0.04	− 0.05	− 0.14^*^	− 0.05	− 0.19^**^	− 0.05	0.01	0.37^**^	− 0.07	1					
**Psychological and Relational Health Outcomes**																	
11. Anxiety	0.56	0.63	0.18^**^	0.22^**^	0.20^**^	0.16^**^	0.35^**^	0.22^**^	0.17^**^	− 0.34^**^	− 0.20^**^	− 0.25^**^	1				
12. Depression	0.68	0.68	0.16^**^	0.22^**^	0.19^**^	0.13^*^	0.34^**^	0.22^**^	0.17^**^	− 0.43^**^	− 0.22^**^	− 0.22^**^	0.89^**^	1			
13. Somatization	0.85	0.66	0.22^**^	0.21^**^	0.24^**^	0.22^**^	0.33^**^	0.33^**^	0.16^**^	− 0.34^**^	− 0.15^*^	− 0.20^**^	0.80^**^	0.73^**^	1		
14. Interpersonal Sensitivity	0.61	0.67	0.19^**^	0.25^**^	0.16^*^	0.15^*^	0.26^**^	0.12^*^	0.10	− 0.39^**^	− 0.26^**^	− 0.04	0.77^**^	0.83^**^	0.62^**^	1	
15. Hostility	0.53	0.58	0.16^**^	0.18^**^	0.26^**^	0.14^*^	0.36^**^	0.16^**^	0.18^**^	− 0.27^**^	− 0.15^*^	− 0.10	0.70^**^	0.74^**^	0.59^**^	0.66^**^	1

Note. **p <.01; *p <.05

Data fully supported H1, revealing that stressors in nursing linked to interpersonal conflict (H1a), inter-role conflicts (H1b) and work-related stress dimensions (H1c) were all significantly positively related to all psychological and relational health outcomes investigated.

Furthermore, all the statistically significant correlations among the study variables provided adequate evidence supporting the mediating and moderating hypotheses testing. Specifically, data firstly revealed that stressors in nursing linked to interpersonal conflict were significantly positively related to the hypothesised mediators, namely work-related stress and inter-role conflict dimensions, i.e., *Conflicts with Physicians* was significantly positively related to both *Work-Family Conflict* (*WFC*) and *Family-Work Conflict* (*FWC*), *Conflicts with Peers* and *Patients and their Families* were significantly positively related to *WFC*, while *Conflicts with Supervisors* was significantly positively related to *Effort-Reward Imbalance* (*ERI*) and to *WFC*.

Moreover, significant correlations between, respectively, stressors in nursing linked to interpersonal conflict, work-related stress and inter-role conflict dimensions with the hypothesised moderators (work-resources) were found, i.e., *Job Control* significantly negatively related to *ERI*, *WFC*, and *FWC; Social Support* significantly negatively related to *ERI*; *Job Satisfaction* significantly negatively related to *Conflicts with Supervisors* and to *ERI*. Additionally, work-resources were also found significantly negatively related to psychological and relational health outcomes.

Furthermore, Skewness values fall within the range of -2 to + 2 (i.e., Skewness values ranged from − 0.44 to + 1.84) and Kurtosis values fall within the range of -7 to + 7 (i.e., Kurtosis values ranged from − 0.86 to + 4.50). Therefore, findings indicated that our data were approximately normally distributed.

### Hypothesis two (H2)– mediating effects

Table 2 displays path coefficients for direct and indirect effects of interpersonal conflicts-stressors in nursing, work-related stress, and work-family inter-role conflicts on psychological and relational health outcomes.

**Table 2:** Path Coefficients: Direct and Indirect Effects

Independent variable	Mediators	Dependent variable	Path A^a^ [95% C.I.]	Path B^b^ [95% C.I.]	Direct effect^c^ [95% C.I.]	Indirect effect^d^ [95% C.I.]	Sobel’s Z^e^
Conflicts	Work-Family	Anxiety^f^	0.40 [0.22, 0.59] ^***^	0.02 [0.01, 0.03] ^**^	02 [0.01, 0.03]^*^	0.01 [0.00, 0.01]^*^	2.45^*^
with	Conflict	Depression^g^	0.40 [0.22, 0.59] ^***^	0.02 [0.01, 0.03] ^**^	0.02 [-0.01, 0.03]	0.01 [0.00, 0.01]^*^	2.48^*^
Physicians		Somatization^f^	0.40 [0.22, 0.59] ^***^	0.03 [0.01, 0.04]^***^	0.02 [0.00, 0.04]^*^	0.01 [0.01, 0.02]^**^	2.92^**^
Conflicts	Work-Family	Anxiety^f^	0.25 [0.07, 0.44] ^**^	0.02 [0.01, 0.03] ^**^	0.02 [0.01, 0.04]^**^	0.01 [0.00, 0.01]^*^	2.01^*^
with	Conflict	Depression^f^	0.25 [0.07, 0.44] ^**^	0.02 [0.01, 0.03] ^**^	0.03 [0.01, 0.04]^**^	0.01 [0.00, 0.01]^*^	2.02^*^
Peers		Somatization^f^	0.25 [0.07, 0.44] ^**^	0.03 [0.01, 0.04]^***^	0.02 [0.01, 0.04]^**^	0.01 [0.00, 0.01]^*^	2.39^*^
	Effort-Reward	Depression^f^	0.01 [0.00, 0.02]^*^	0.49 [0.31, 0.66] ^***^	0.01 [0.00, 0.03]^*^	0.01 [0.00, 0.01] ^*^	2.22^*^
	Imbalance	Somatization^f^	0.01 [0.00, 0.02]^*^	0.45 [0.28, 0.62] ^***^	0.02 [0.01, 0.03]^***^	0.01 [0.00, 0.02] ^*^	2.21^*^
Conflicts		Interpersonal-Sensitivity^f^	0.01 [0.00, 0.02]^*^	0.36 [0.18, 0.54] ^***^	0.02 [0.00, 0.02]^*^	0.01 [0.00, 0.01] ^*^	2.06^*^
with		Hostility^f^	0.01 [0.00, 0.02]^*^	0.42 [0.27, 0.57] ^***^	0.02 [0.01, 0.03]^***^	0.01 [0.00, 0.01] ^*^	2.24^*^
Supervisors							
	Work-Family	Anxiety^f^	0.26 [0.12, 0.39]^***^	0.02 [0.01, 0.03] ^**^	0.02 [0.00, 0.03]^**^	0.01 [0.00, 0.01] ^*^	2.30^*^
	Conflict	Depression^f^	0.26 [0.12, 0.39]^***^	0.02 [0.01, 0.03] ^**^	0.02 [0.00, 0.03]^**^	0.01 [0.00, 0.01] ^*^	2.34^*^
		Somatization^f^	0.26 [0.12, 0.39]^***^	0.03 [0.01, 0.04] ^***^	0.02 [0.00, 0.03]^**^	0.01 [0.00, 0.01] ^**^	2.92^**^
Patients	Work-Family	Anxiety^g^	0.23 [0.11, 0.34] ^***^	0.02 [0.01, 0.03] ^**^	0.01 [-0.00, 0.02]	0.01 [0.00, 0.01] ^**^	2.41^*^
and	Conflict	Depression^g^	0.23 [0.11, 0.34] ^***^	0.02 [0.01, 0.03] ^**^	0.01 [-0.00, 0.02]	0.01 [0.00, 0.01] ^**^	2.48^*^
their		Somatization^f^	0.23 [0.11, 0.34] ^***^	0.03 [0.01, 0.04] ^***^	0.01 [0.00, 0.02] ^**^	0.01 [0.00, 0.01] ^**^	3.06^**^
Families		Hostility^g^	0.23 [0.11, 0.34] ^***^	0.03 [0.01, 0.04] ^***^	0.01 [-0.00, 0.02]	0.01 [0.00, 0.01] ^*^	1.98^*^

*Note* Only significant mediation models were displayed

^a^Path A, Effect of independent variable on mediator; ^b^Path B, Effect of mediator on dependent variable; ^c^Direct Effect, Effect of independent variable on dependent variable controlling for the mediator; ^d^Indirect Effect, Effect of independent variable on dependent variable through the mediator; ^e^Sobel’s *Z*, Sobel test results for indirect effect; ^f^Partial Mediation, ^g^Full Mediation. *p <.05. **p <.01. ***p <.001

Specifically, data revealed that all the stressors in nursing linked to interpersonal conflicts were able to significantly exacerbate perceived levels of *WFC* (H2a). while only *Conflicts with Supervisors* was able to significantly exacerbate perceived levels of *ERI* (H2b).

**Fig. 2:**
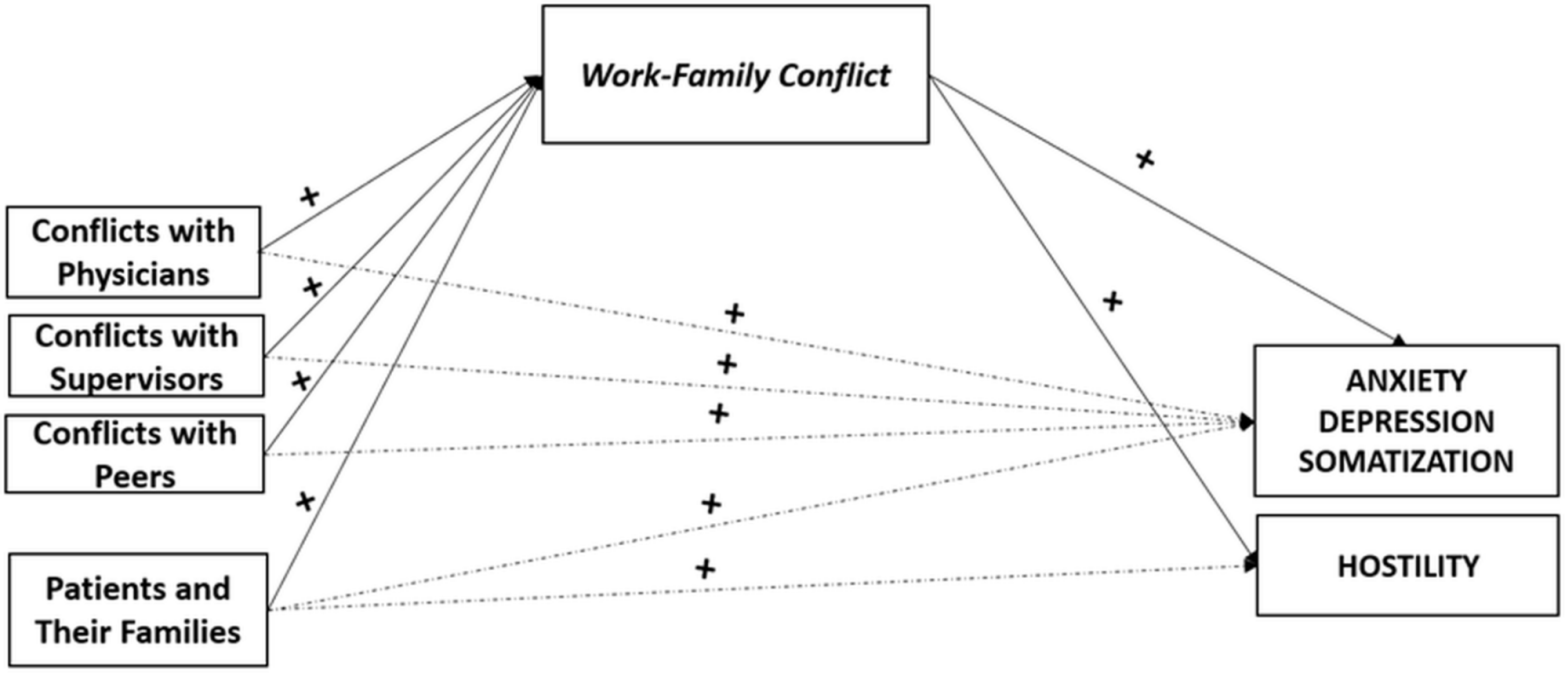
Summary: the mediating role of Work-Family Conflict. *Note.* Mediating variables are displayed in italics; Health outcomes are displayed in capital. Symbols (+) indicate the directions of the associations

Also, overall data supported the mediating role of *ERI* and of *WFC*– yet no evidence were provided for the mediating role of *FWC*. In particular, data revealed that *WFC* (H2c) played as a statistically significant mediator in the associations between all stressors in nursing linked to interpersonal conflicts and, respectively Anxiety, Depression and Somatization. Furthermore, *WFC* also played as statistically significant mediator in the association between the stressor linked to interpersonal conflicts with *Patients and Their Families* and Hostility (Fig. 2).

**Fig. 3:**
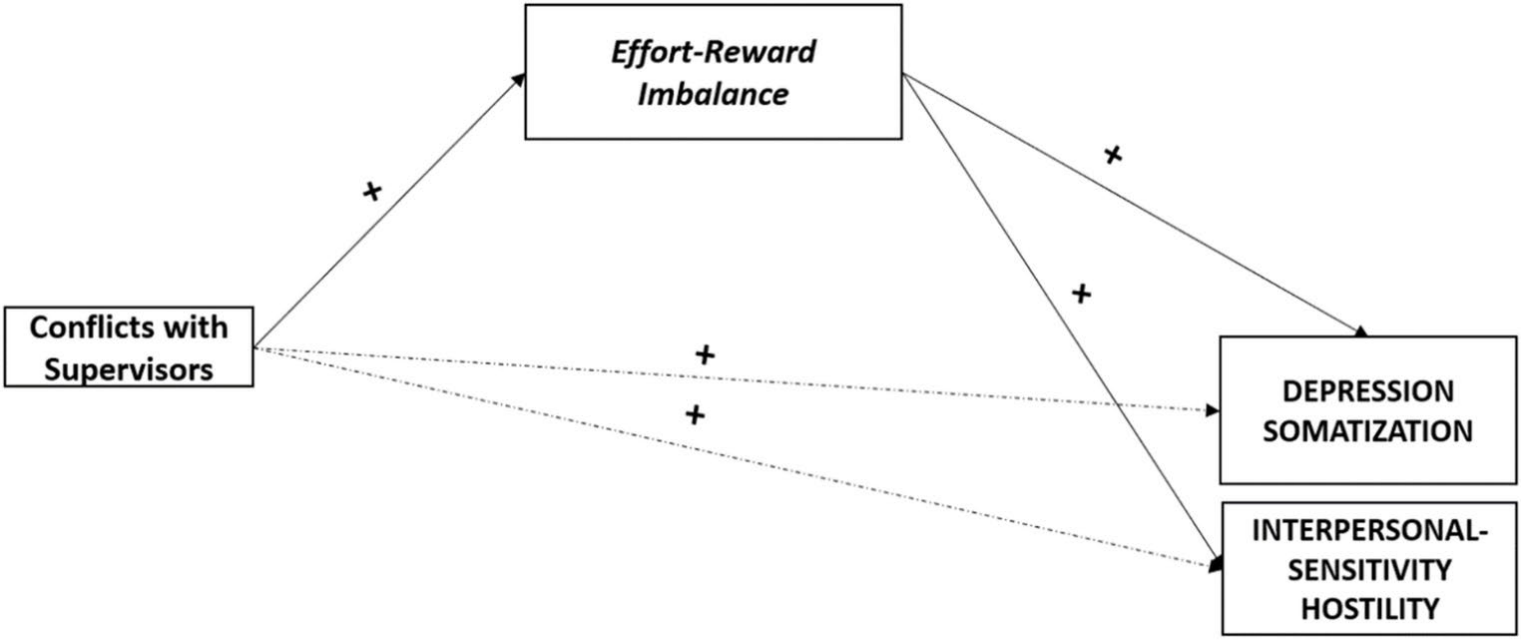
Summary: the mediating role of Effort-Reward Imbalance. *Note.* Mediating variables are displayed in italics; Health outcomes are displayed in capital. Symbols (+) indicate the directions of the associations

Moreover, data revealed that *ERI* (H2d) played as a statistically significant mediator in the associations between *Conflicts with Supervisors* and, respectively, Anxiety, Depression, Interpersonal-Sensitivity, and Hostility (Fig. 3). Additionally, when checking for multicollinearity, the Variance Inflation Factors (VIFs) values for the models tested were all < 5 (*range* 1.02 to 1.07) and the tolerance values were all > 0.40 (*range* 0.93 to 0.97), supporting the significant role of all the dimensions considered.

### Hypothesis three (H3)– moderating effects

Data fully supported the significant moderating role of all Work Resources (H3), also highlighting some specificities.

**Fig. 4:**
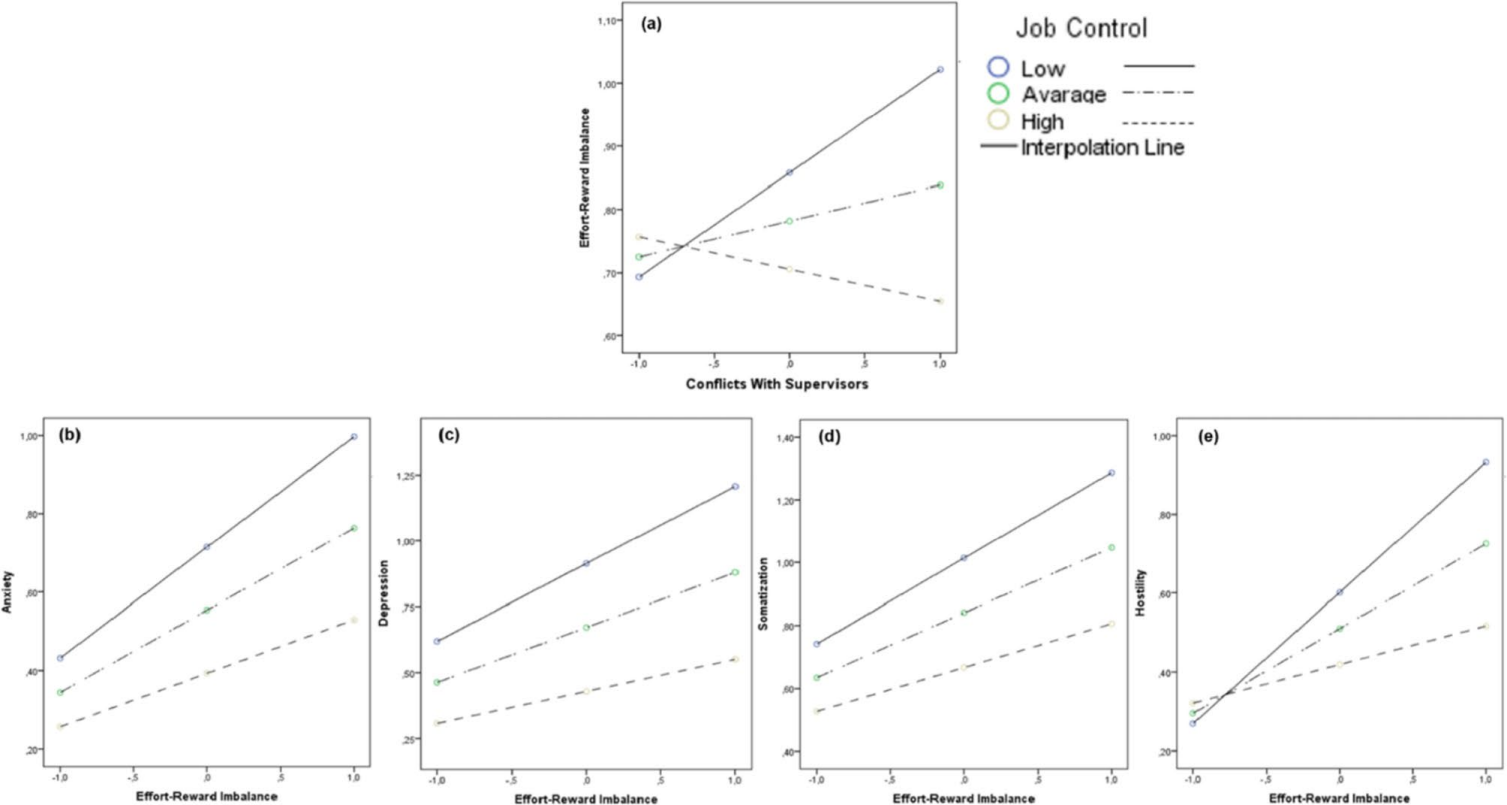
The moderating role of Job Control. *Note.* The moderating role of Job Control in the associations between Conflicts With Supervisors and Effort-Reward Imbalance **(a)**, and in the associations between Effort-Reward Imbalance and Anxiety **(b)**, Depression **(c)**, Somatization **(d)**, and Hostility **(e)**

Firstly, data revealed the statistically significant moderating effect of perceived *Job Control* (H3a), specifically in the associations between *Conflicts with Supervisors* and *ERI* (Conflicts with Supervisors × Job Control against ERI: ΔR^2^ = 0.060, *t* = -4.17, *p* =.000), as well as in the associations between *ERI* and, respectively, Anxiety (ERI × Job Control against Anxiety: ΔR^2^ = 0.020, *t* = -2.65, *p* =.008), Depression (ERI × Job Control against Depression: ΔR^2^ = 0.024, *t* = -2.98, *p* =.003), Somatization (ERI × Job Control against Somatization: ΔR^2^ = 0.016, *t* = -2.27, *p* =.024), and Hostility (ERI × Job Control against Hostility: ΔR^2^ = 0.063, *t* = -4.65, *p* =.000) (Fig. 4).

**Fig. 5:**
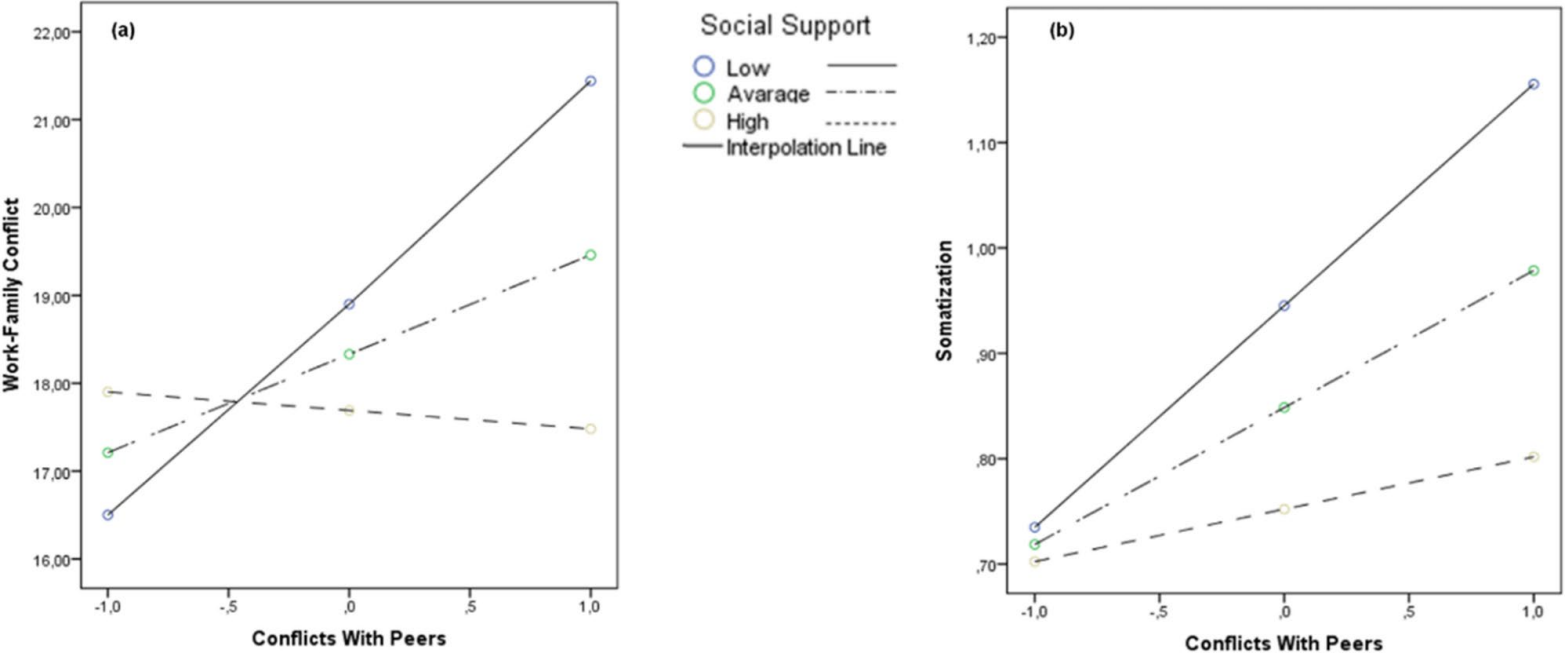
The moderating role of Social Support. *Note.* The moderating role of Social Support in the associations between Conflicts with Peers and, respectively, Work-Family Conflict **(a)** and Somatization **(b)**

Secondly, data supported the statistically significant moderating effect of perceived *Social Support* (H3b), specifically in the associations between *Conflicts with Peers* and, respectively, *WFC* (Conflicts with Peers × Social Support against Work-Family Conflict: ΔR^2^ = 0.080, *t* = -2.86, *p* =.004) and Somatization (Conflicts with Peers × Social Support against Somatization: ΔR^2^ = 0.015, *t* = -2.03, *p* =.043) (Fig. 5).

**Fig. 6:**
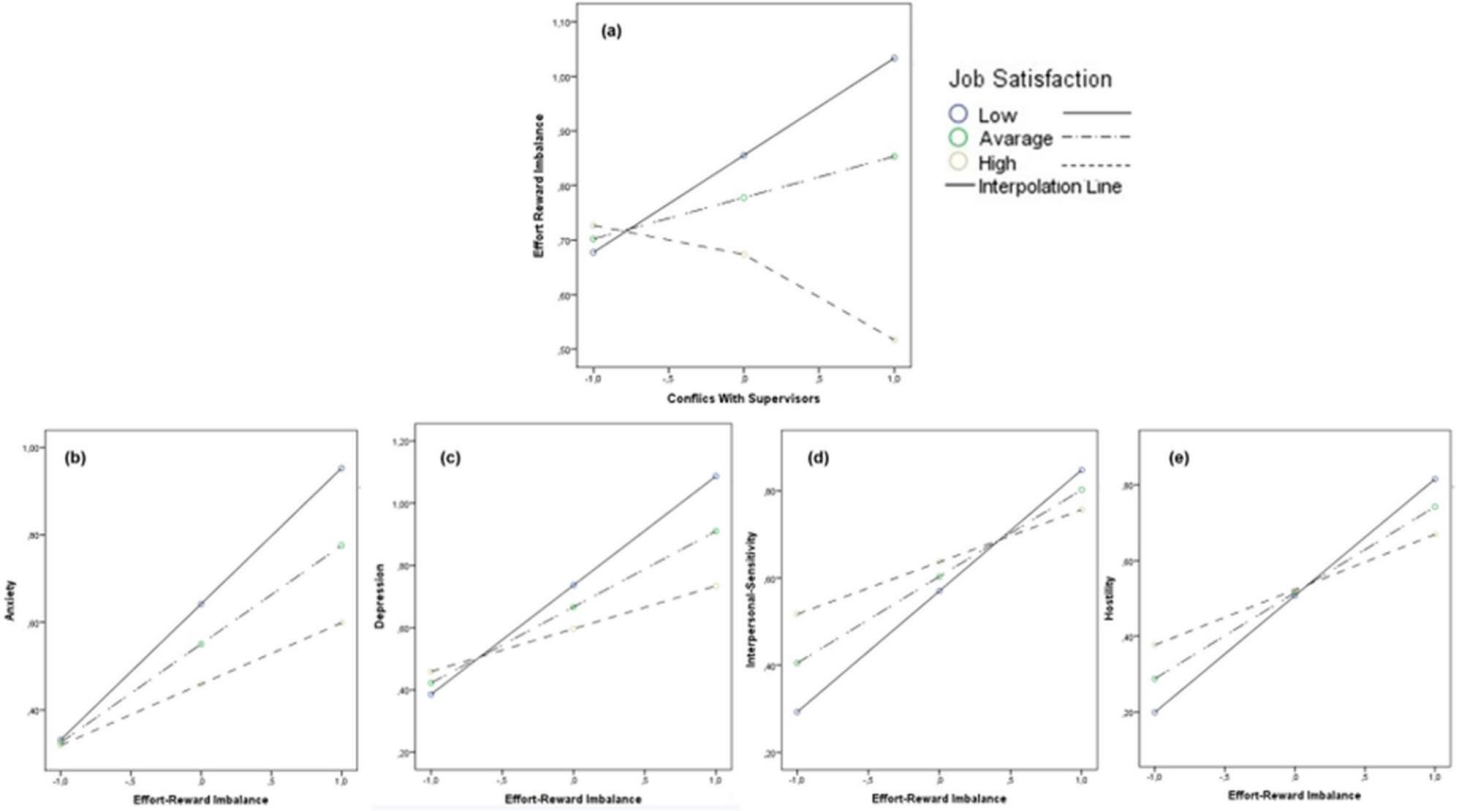
The moderating role of Job Satisfaction. *Note.* The moderating role of Job Satisfaction in the associations between Conflicts with Supervisors and Effort-Reward Imbalance **(a)** and in the associations between Effort-Reward Imbalance and Anxiety **(b)**, Depression **(c)**, Interpersonal-Sensitivity **(d)**, and Hostility **(e)**

Finally, data highlighted the statistically significant moderating role of Job Satisfaction (H3c). Specifically, data revealed the moderating role of Job Satisfaction in the associations between *Conflicts With Supervisors* and *ERI* (Conflict With Supervisors × Job Satisfaction against ERI: ΔR2 = 0.057, *t* = -4.07, *p* =.000), as well as in the associations between *ERI* and, respectively, Anxiety (ERI × Job Satisfaction against Anxiety: ΔR^2^ = 0.036, *t* = -3.40, *p* =.000), Depression (ERI × Job Satisfaction against Depression: ΔR^2^ = 0.046, *t* = -3.85, *p* =.000), Interpersonal-Sensitivity (ERI × Job Satisfaction against Interpersonal-Sensitivity: ΔR^2^ = 0.026, *t* = -2.76, *p* =.006), and Hostility (ERI × Job Satisfaction against Hostility: ΔR^2^ = 0.038, *t* = -3.45, *p* =.000) (Fig. 6).

**Fig. 7:**
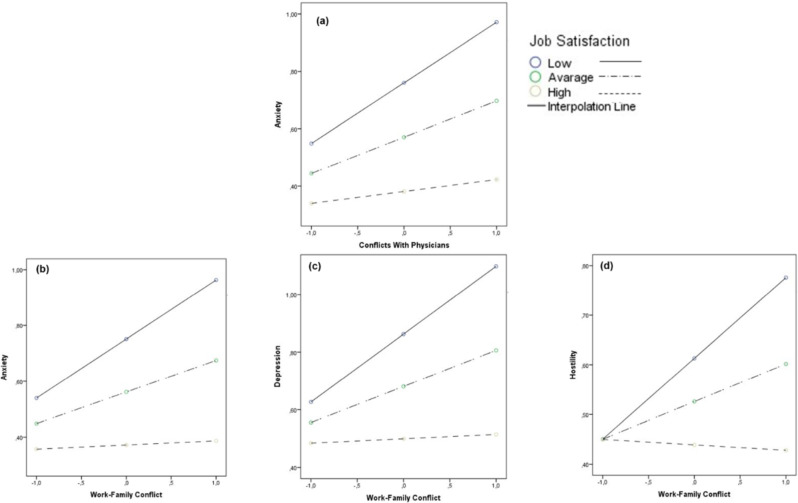
The moderating role of Job Satisfaction. *Note.* The moderating role of Job Satisfaction in the associations between Conflicts with Physicians and Anxiety **(a)** and in the associations between Work-Family Conflict and Anxiety **(b)**, Depression **(c)**, and Hostility **(d)**

Furthermore, Job Satisfaction was found able to also significantly moderate the associations between *Conflicts with Physicians* and Anxiety (Conflicts with Physicians × Job Satisfaction against Anxiety: ΔR^2^ = 0.018, *t* = -2.30, *p* =.021), as well as the associations between *WFC* and, respectively, Anxiety (Work-Family Conflict × Job Satisfaction against Anxiety: ΔR^2^ = 0.023, *t* = -2.62, *p* =.009), Depression (Work-Family Conflict × Job Satisfaction against Depression: ΔR^2^ = 0.024, *t* = -2.66, *p* =.008), and Hostility (Work-Family Conflict × Job Satisfaction against Hostility: ΔR^2^ = 0.021, *t* = -2.41, *p* =.016) (Fig. 7).

No other significant moderating effects were found. Additionally, the Variance Inflation Factors (VIFs) values for the models tested were all < 5 (*range* 1.00 to 1.04) and the tolerance values were all > 0.40 (*range* 0.96 to 0.99), supporting the significant role of all the dimensions considered.

## Discussion

The present study proposed a research application of the *DRIVE-Nurses Model* [9] with the aim of responding to the need to go in-depth into the complex and dynamic interplay between stress, conflict and psychological/relational health conditions among nurses. This while acknowledging, at the same time, the reciprocity of nurses’ experiences within and outside the wards.

Firstly, findings highlighted alarming levels of psychological suffering among sampled nurses, given the relevant number of nursing staff overwhelming the clinical cut-off scores for symptoms of Somatization (18.5%), Depression (15.5%), and Anxiety (14.0%). Moreover, data suggested the presence of relevant levels of relational suffering, given the number of nurses overwhelming the clinical cut-off scores for symptoms of Hostility (11.7%) and, even more, for Interpersonal-Sensitivity (18.1%), the latter featured by negative expectations concerning relationships, perceived low esteem from others, low self-evaluation, and sense of inferiority. These findings should be carefully considered, given that nurses’ psychological and relational discomfort may, in turn, play a role in exacerbating perceived levels of stress and in escalating interpersonal conflicts both in healthcare settings and in private life.

Accordingly, overall, findings provided initial evidence sustaining the risks of vicious circles featured by the exacerbation of work-related stress, interpersonal and inter-role conflicts, as well as psychological and relational suffering (H1). Specifically, in line with previous research [4, 17, 34–36, 41, 50, 51], findings highlighted the detrimental impact of perceived stress and conflicts within the work domain and beyond the healthcare setting at the individual level, in terms of increasing symptoms of Anxiety, Depression, and Somatization. However, data also confirmed the relational risks nurses are exposed at [43], given the link between perceived stress and conflicts also with nurses’ symptoms of Hostility and Interpersonal-Sensitivity. Furthermore, our first data also sustained previous research which highlighted the need to focus not only on relationships with patients and their relatives [29, 78], but also on the relationships with co-workers - other nurses, physicians, supervisors [47, 56, 63,, 79, 80], which, indeed, may also represent significant sources of stress and conflict to be addressed.

However, when going in-depth into the relationships among study variable to test mediating effects, more complex interplay dynamics were found (H2). Firstly, data suggested that nurses who perceived higher levels of stress linked to conflicts with physicians, supervisors and peers, along with those who perceived stress in the relationships with patients and their families were likely to report escalating perceived inter-role conflict, in terms of work-family conflict - yet not in terms of family-work conflict. These results supported previous research highlighting the ability of experiences in workplace (conflict/violence) to *crossover* the work domain [15, 16], exacerbating conflict and suffering nurses may experience in private life [4, 53].

Nevertheless, the *non-significance* of the associations between stressors in nursing linked to interpersonal conflict and family-work conflict (i.e., family interfering with work) seems suggesting that work life is able to interfere with private life to a greater extent than *viceversa*. This could be linked to the inherent characteristic of nursing profession, which is indeed a “shift work”, so that nurses may be used to plan/schedule their private life based on their work-shift to a greater extent than *viceversa*. However, overall, these findings supported the meaningful to develop interventions, programmes and campaigns promoting psychological/relational health among healthcare staff by starting from the workplaces, yet potentially having a positive impact also on nurses’ private life.

Moreover, data also enlightened the significant mediating role of work-family conflict, with all stressors in nursing linked to interpersonal conflicts having an impact in terms of increasing anxiety, depression, and somatization also through the exacerbation of perceived inter-role conflict. However, a tailored attention should be given to perceived stress linked to problems and conflicts in the relationships with patients and their families. Indeed, data suggested a specific spiral effect of stress and conflict, in which the interplay between perceived stress linked to conflict with patients/their family and work-family conflict have a detrimental impact increasing nurses’ symptoms of hostility. In other words, data may highlight a vicious circle in which the anger and the frustration experienced in the relationship with “clients” may exacerbate the anger and the frustration experienced outside the wards, and these dynamics may significantly impair and deplete nurses’ relational skills, making more likely the possibility of emergencies of conflicts as well as their escalation in both work and life domains.

However, when considering stress linked to perceived effort-reward imbalance, our data highlighted that only the relationships with supervisors [47, 56, 81] may play a pivotal role in exacerbating perceived mismatch between high efforts spent at work and low rewards (esteem and material) received at work, determining individual and relational disease. These findings provided further evidence endorsing the idea of perceived workplace conflicts being able to exacerbate perceived levels of work-related stress [33, 54–56], yet also added tailored information on those actors/relationships– in such case with supervisors - that should be carefully considered when defining interventions reducing perceived effort-reward imbalance and promoting occupational health among nurses.

In this direction, when testing for the moderating effects, findings allowed the identification of specific moderating variables (i.e., job control; social support; job satisfaction) that should be targeted and promoted within interventions, as they were able to significantly buffer the negative impact of nearly all sources of stress and conflicts (all but patients and their families), potentially breaking the abovementioned vicious circles. In particular, in line with research supporting the significant role of perceived job control as key resources within work domains [57, 58], data revealed that perceived Job Control (perceived skill discretion and decision authority) was able not only to prevent perceived *Conflicts with Supervisors* from exacerbating perceived Effort-Reward Imbalance, but also to reduce the risk of individual and relational suffering.

Differently, perceived Social Support was found able to significantly prevent *Conflicts with peers* from cross-overing the wards, exacerbating nurses’ perceived levels of *Work-Family Conflict* and increasing psychophysical disease. These latter findings were in line with research highlighting the meaningful role of support within work-context [59–61], but also provided further evidence supporting how this important resource may promote high-quality relationships among co-workers.

Last, but not least, data highlighted the pivotal moderating role of Job Satisfaction [62, 63], which was found able to significantly buffer the negative impact of *Conflicts with Supervisors* and *Conflicts with Physicians*, along with the negative impact of *ERI* and *WFC* on psychological and relational health conditions. These data emphasised the need to carefully assess and promote self-accomplishment, which, indeed, may undoubtedly drive the conditions fostering greater work life and even general life experiences.

In conclusion, whereas perceived stress and conflicts are parts of everyday life in the workplace and in the private domains, our findings demonstrated how they can escalate and result in vicious circles exacerbating not only individual suffering (anxiety, depression, somatization), but also relational disease, with increasing interpersonal sensitivity and hostility undermining the quality of all relations within and outside the workplace. Nonetheless, our data also suggested that providing and fostering work resources such job control, social support, and– above all– job satisfaction can effectively break these vicious circles, so promoting individual and relational wellbeing among nurses.

Despite its merits, the study has some limitations that should be addressed. Firstly, one limitation is the cross-sectional design, so that data can only provide a temporary picture of these complex dynamics, causality cannot be conclusively determined, nor can the direction of effects be established. A longitudinal design would provide stronger evidence for the unveiled dynamics and would allow suggesting cause-effect/temporal relationships between predictors and outcomes. Therefore, future research is recommended to be developed with a longitudinal design - so assessing and monitoring psychological and relational health conditions within the wards over time - yet also intervention studies should be considered. Indeed, new findings of such research design (assessing study variables pre-to-post interventions) could provide direct evidence of how changes in perceived stressors and resources may affect psychological and relational health outcomes. This would give further and tailored indications to be used by the hospital management in designing evidence-based programs and interventions. Secondly, data relies on participants’ self-reports; therefore, findings could be affected by the risk of social desirability bias as well by the underestimation/overestimation of perceived stress and conflicts by nurses. Indeed, despite we have focused on perceived conflicts rather than on episodes of violence to reduce the perceived stigma of victimization (i.e., shame, fear of judgement, blame/non-supportive environment) and fear of consequences for their selves [22–26], we cannot guarantee that interpersonal conflicts were underestimated and believed as not enough serious be reported. In line with this, further research could also be designed to include a wider range of sources of data. Specifically, the collection of qualitative data could enrich the understanding of the nuanced experiences of nurses dealing with stress and conflicts, also providing further evidence of how they perceive the support and resources available to them. Thirdly, although findings could be of international interest, the study offered original evidence on nurses working in the Italian healthcare context. Therefore, future studies could be developed with a cross-cultural design to test the generalizability of our results. In the same direction, future research could consider the inclusion of further factors that could be able to break vicious circles, in particular considering that our data provided evidence for the moderating role of work-resources in counteracting the negative impact of all detrimental factors but *Patients and Their Families*. From this perspective, our results do not fully align with previous research supporting the moderating role of work resources, in particular social support, in the context of workplace violence against/between nurses. However, to the best of our knowledge, research testing the potential role of moderating variables in this context is still scarce, and there is still lack of consensus in the measurement tools used for assessing conflict/violence, work-resources, and outcomes. Accordingly, whereas this discrepancy could be the result of several factors, including cultural differences and specificities of the Italian healthcare system, this could be also due to the different ways the topic– of international interest– has been treated in research (i.e., differences in study variables and measures used) [34]. For example, there are several ways/tools in the literature to assess perceptions of violence/conflict by distinguishing perpetrators (e.g., internal/external violence [59], users’ violence [63]), but the topic is also explored by assessing the frequency of experiences of physical/psychological violence at work beyond perpetrators [61]. Also, some studies targeting workplace conflict/violence have provided evidence supporting the moderating role of work resources concerning outcomes such as perceived job insecurity [57] and turnover intentions [59, 60, 62]. Other research in the field explored psychological wellbeing as the outcome, yet they considered psychological outcomes and tools that varied from those adopted in the present study (e.g., emotional well-being, fear of future violence at work, job-related affect [61], burnout [62, 63]). Further evidence and comparative research in the field, mainly those exploring mediating/moderating effects, are therefore needed to shed light on and endorse this initial evidence on vicious and virtuous circles.

However, despite these limitations, our data unveiled the risk of a vicious circles of stress, conflicts and violence, extremely harmful for nurses’ wellbeing, since increasing anger and frustration, exacerbating negative interactions, communication gaps, and the risk of reporting poor mental health. Therefore, helping and improving a virtuous relational approach, developing evidence-based programmes and interventions fostering work resources and also involving supervisors, physicians, patients, and their families, should be considered one of the main goals for healthcare organizations who wish to effectively promote nurses’ wellbeing. Specifically, hospital managers and relevant stakeholders in public health could consider findings from the present study to develop and/or enhance programs for routinely assessing and monitoring perceived stress, conflicts, and psychological/relational health within the wards, so timely implementing evidence-based interventions preventing disease and suffering escalations.

With particular reference to conflicts and psychological/relational suffering (i.e., potential vicious circles), specific information and mandatory training on the relevant detrimental impact of non-physical abuses (acknowledging all the actors involved within and beyond the wards), and on the importance of reporting and seeking professional/institutional support - guaranteeing help and understanding as well as actions for the perpetrators - should be widely offered to the whole healthcare staff. The work-family spillover perspective (conflict/enrichment) should be also acknowledged and recognized (i.e., potential vicious and virtuous circles), and the healthcare staff should be offered the possibility to access psychological services within all the hospitals.

Tailored training on stress, conflicts, and ways/specific resources to be enhanced for dealing with them effectively should be also developed and offered routinely to hospital managers and the whole healthcare staff. This is also by taking into account that dealing with conflicts and stress effectively would promote a better and satisfactory work climate in the wards, resulting in high-quality performance and standards of care provided by the healthcare staff.

### Electronic supplementary material

Below is the link to the electronic supplementary material.

Supplementary Material 1

## Data Availability

The datasets generated and/or analyzed during the current study are not publicly available due to the terms of consent to which the participants agreed but available from the corresponding author on reasonable request. No datasets were generated or analysed during the current study.

## Abbreviations

ERI: Effort-Reward Imbalance
DRIVE Model: Demands-Resources and Individual Effects Model
FWC: Family-Work Conflict
WFC: Work-Family Conflict
