# Supplementary material for: Stress, interpersonal and inter-role conflicts, and psychological health conditions among nurses: vicious and virtuous circles within and beyond the wards
Source: BMC Psychol. 2024 Apr 10;12:197. doi: 10.1186/s40359-024-01676-y (PMC11007966; doi:10.1186/s40359-024-01676-y)
Supplement: Supplementary file 1 — Supplementary Material 1 [file 40359_2024_1676_MOESM1_ESM.docx]

**Supplementary Table 1.** Cronbach’s α and McDonald’s ω values for study variables

|  |  | *Cronbach’s* | McDonald’s |
| --- | --- | --- | --- |
|  |  | *α* | *ω* |
| **Interpersonal Conflicts**  **Stressors in Nursing** |  |  |  |
| Conflicts with Physician |  | .796 | .801 |
| Conflicts with Peers |  | .839 | .861 |
| Conflicts with Supervisors |  | .819 | .824 |
| Patients and their Families |  | .865 | .861 |
| **Work-Related Stress** |  |  |  |
| Effort-Reward Imbalance- Efforts |  | .815 | .816 |
| Effort-Reward Imbalance-Total Rewards |  | .842 | .839 |
| **Work-Family Inter-role Conflicts** |  |  |  |
| Work-Family Conflict |  | .887 | .888 |
| Family-Work Conflict |  | .886 | .886 |
| **Work Resources** |  |  |  |
| Job Control |  | .761 | .771 |
| Social Support |  | .804 | .812 |
| Job Satisfaction |  | .897 | .899 |
| **Psychological and Relational Health Outcomes** |  |  |  |
| Anxiety |  | .876 | .886 |
| Depression |  | .905 | .913 |
| Somatization |  | .869 | .875 |
| Interpersonal Sensitivity |  | .857 | .868 |
| Hostility |  | .788 | .791 |
